# Blood nerve barrier permeability enables nerve targeting of circulating nanoparticles in experimental autoimmune neuritis

**DOI:** 10.1038/s41598-025-96231-z

**Published:** 2025-04-06

**Authors:** Chanpreet Kaur, Ellaina Villarreal, Maleen H. Cabe, Kelly A. Langert

**Affiliations:** 1 Department of Molecular Pharmacology and Neuroscience, Stritch School of Medicine, Loyola University Chicago, 2160 S. First Avenue, Bldg 115, Room 416, Maywood, IL 60153 USA; 2 Research Service, Edward Hines Jr. VA Hospital, Hines, IL 60141 USA

**Keywords:** Peripheral nerve, Nanoparticle, Blood-nerve barrier, Experimental autoimmune neuritis, Delivery, Peripheral nervous system, Drug delivery, Diseases of the nervous system

## Abstract

Guillain-Barré syndrome (GBS) is a devastating autoimmune disease of the peripheral nervous system (PNS) with limited treatment options. Several studies have shown attenuation of the well-characterized GBS preclinical experimental autoimmune neuritis (EAN) model with systemically administered therapeutic compounds via anti-inflammatory or immunomodulatory mechanisms. Despite this, clinical advancement of these findings is limited by dosing that is not translatable to humans or is associated with off-target and toxic effects. This is due, in part, to the blood-nerve barrier (BNB), which restricts access of the circulation to peripheral nerves. However, during acute neuroinflammation, the normally restrictive BNB exhibits increased vascular permeability and enables immune cell infiltration. This may offer a unique window to access the otherwise restricted peripheral nerve microenvironment for therapeutic delivery. Here, we assessed the degree to which BNB permeability and immune cell infiltration over the course of EAN enables accumulation of circulating nanoparticles. We found that at disease stages defined by distinct clinical scores and pathology (onset, effector phase, and peak of EAN severity), intravenously administered small molecules and nanoparticles ranging from 50 to 150 nm can permeate into the endoneurium from the endoneurial vasculature in a size- and stage-dependent manner. This permeation occurs uniformly in both sciatic nerves and in proximal and distal regions of the nerves. We propose that this nerve targeting enabled by pathology serves as a platform by which potential therapies for GBS can be reevaluated and investigated preclinically in nanoparticle delivery systems.

## Introduction

Guillain–Barre syndrome (GBS) is a devastating autoimmune disease of the peripheral nervous system (PNS) for which there is no targeted therapy^1,2^. Experimental autoimmune neuritis (EAN) is a well-established preclinical model of the acute inflammatory demyelinating polyneuropathy (AIDP) subtype of GBS that has advanced our understanding of mechanism by which pathology develops and progresses^3–5^. Several previous studies have shown attenuation of EAN with systemic administration of potential therapeutic compounds that span a range of anti-inflammatory or immunomodulatory strategies^6–11^. While shedding light on the mechanisms of disease, clinical advancement of these findings is limited by dosing that is not translatable to humans or is associated with off-target and toxic effects.

Anatomical features of the peripheral nerves, including compartmentalization by connective tissue and the blood nerve barrier (BNB), collectively represent an obstacle to delivering therapeutics to peripheral nerves^12^. The BNB consists of specialized microvascular endothelial cells linked by tight junctions, creating an isolated endoneurial microenvironment. Several studies have investigated the role of the BNB in restricting access of circulating molecules to the endoneurium under physiological conditions^13^. During acute neuroinflammation, such as that associated with AIDP, the normally restrictive BNB exhibits increased vascular permeability and enables immune cell infiltration^14,15^. While these pathological changes contribute to disease, they may also offer a unique window of opportunity to access the otherwise restricted peripheral nerve microenvironment for therapeutic delivery.

It is established that increases in vascular permeability, such as that occurring at sites of localized inflammation, can promote accumulation of circulating nanoparticles (NPs) with favorable size and surface properties^16–18^. This phenomenon is dependent on prolonged circulation and avoidance of clearance, which are best achieved by NPs in the range of 50–200 nm^19^. Despite promising preclinical results for enhanced permeation and retention (EPR) to promote accumulation of NPs in tumors^20^, successful translation of EPR for chemotherapeutic delivery has been low^21,22^. Established contributing factors include properties and kinetics of tumor development in preclinical models poorly reflect human pathology and tumor microenvironments contain both avascular regions and tortuous vessels, resulting in heterogenous NP distribution^21,22^. Given the established validity of the EAN model and linearity of peripheral nerve vasculature^15,23^, the potential for EPR to facilitate delivery to peripheral nerves in AIDP is an exciting area for preclinical investigation^18^.

In this study, we assessed whether the BNB permeability and immune cell infiltration over the course of EAN enable nerve accumulation of circulating nanoparticles. We identified stages of EAN, termed onset, effector phase, and peak severity, defined by distinct clinical scores and pathology. We found that intravenously administered small molecules (69 kDa) and NPs ranging from 50 to 150 nm can permeate into the endoneurium from the endoneurial vasculature in a size- and disease stage-dependent manner. We propose that this nerve targeting enabled by pathology serves as an opportunity by which potential therapies for GBS can be reevaluated and investigated preclinically in NP delivery systems.

## Results

### Experimental autoimmune neuritis clinical and pathological presentation

**Fig. 1:**
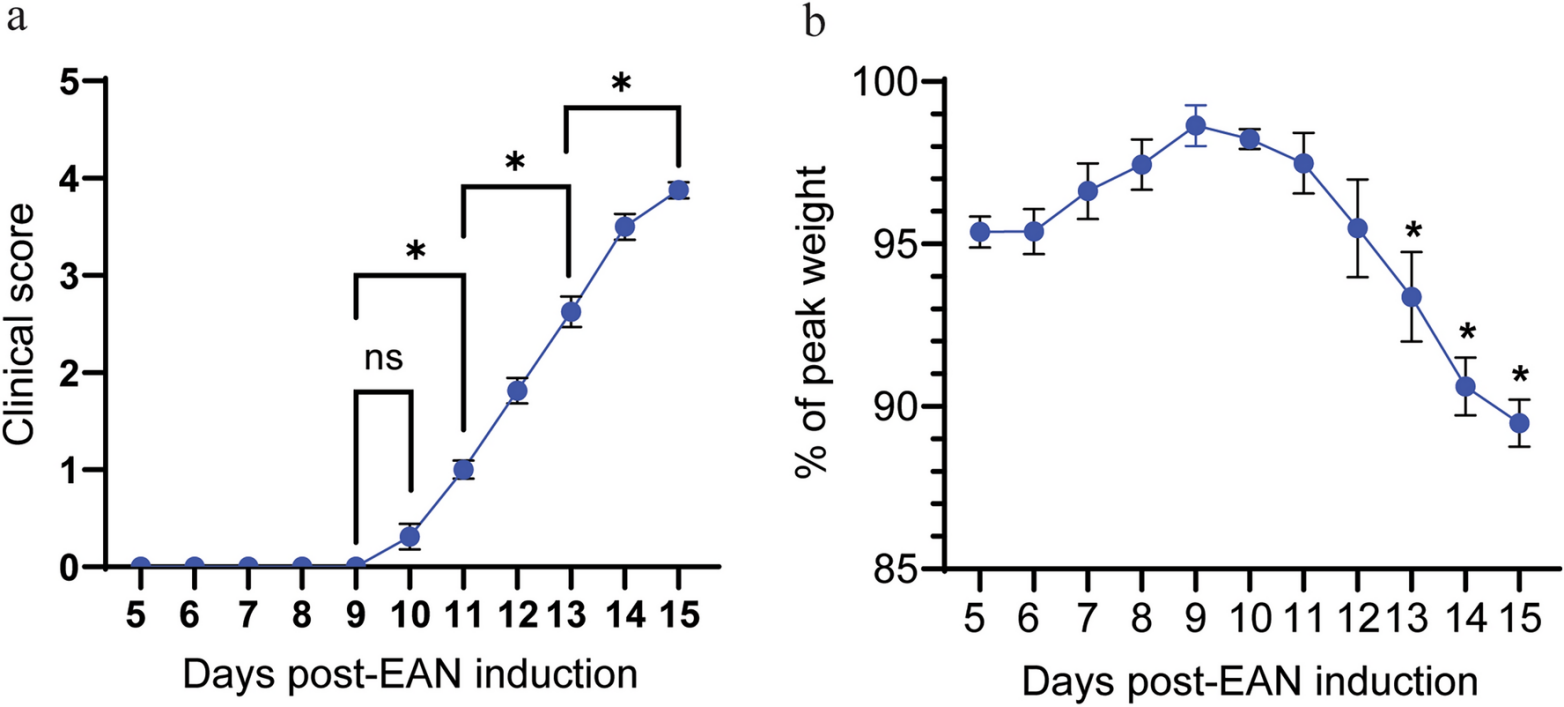
EAN clinical presentation**.** EAN was induced with P2 peptide (residues 53–78) and complete Freund’s adjuvant on Day 0. (**a**) Daily clinical scores of naïve control and EAN rats. Key disease stages include Day 11 (onset), Day 13 (effector phase), and Day 15 (peak severity). (**b**) Daily weights of naïve control and EAN rats, as a percentage of maximum weight. Data shown are the mean ± SEM, n = 8, *p < 0.05, Ordinary one-way ANOVA with Tukey’s multiple comparisons test.

We and others have previously shown that EAN induced with complete Freund’s adjuvant (CFA) and P2 peptide manifests as a robust, monophasic course of ascending weakness^24,25^. Here, the observed disease course develops and progresses similarly, with onset on day 11 post induction presenting as loss of tail tone and the peak of disease severity on day 15 post induction (Fig. 1a). Rats lose weight over the course of EAN, with a significant reduction compared to their maximum weight beginning on Day 13 (Fig. 1b). In this study, we focus on three distinct stages of disease: EAN onset (Day 11), effector phase of EAN (Day 13), and peak severity of EAN (Day 15). The observed clinical scores (Fig. 1a) at each of these stages are distinct and significantly different from baseline and from each other (p < 0.05).

**Fig. 2:**
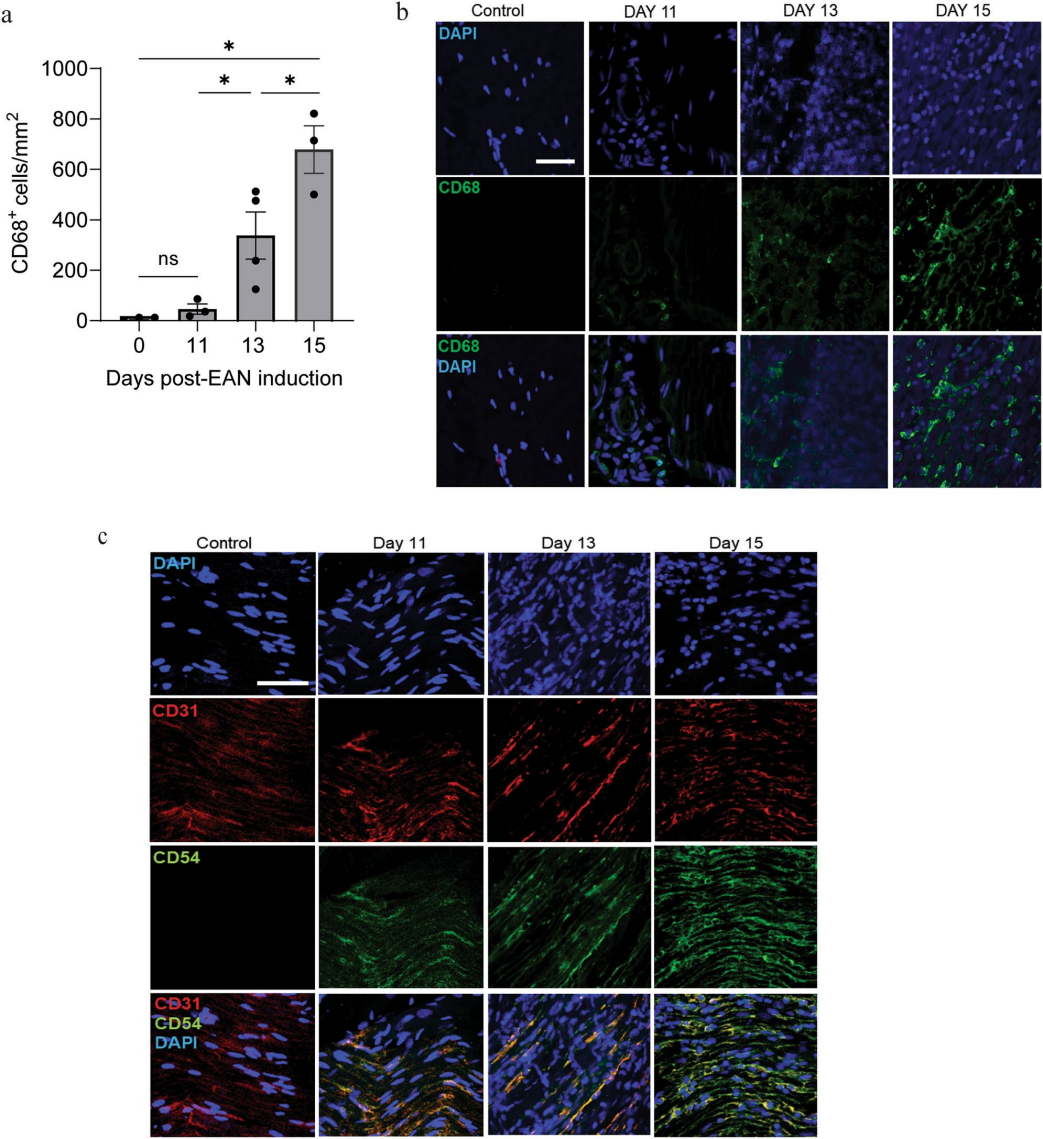
Cellular changes in nerves over the course of EAN**.** Sciatic nerves were harvested at key disease stages and immune and endothelial cells were assessed with immunohistochemistry. (**a**) Quantification of CD68^+^ macrophages in transverse sections. Data shown are the mean ± SEM, n = 3–4 rats, *p < 0.01, one-way ANOVA followed by Fisher’s LSD multiple comparison. (**b**) Representative images of data quantified in (**a**). Green, CD68; blue, DAPI; scale bar, 40 µm. (**c**) Representative longitudinal sections stained for the presence of intercellular adhesion molecule (ICAM)-1 (CD54) and endothelial marker CD31. Green, CD54; red, CD31; blue, DAPI; scale bar, 40 µm.

It is well established that immune cells, including CD68^+^ macrophages, infiltrate nerves at the peak of EAN^5,11,24^. Here, to better understand the ability of immune cells to gain access to the endoneurium at different stages of disease, we quantified CD68^+^ macrophages in cross sections of sciatic nerves collected from animals at onset, effector phase, and peak severity of EAN. Nerves from naïve animals contain a limited number of macrophages (7.9 ± 4.0 cells per mm^2^, Fig. 2a). In the effector phase of EAN, nerves contain a significant increase in macrophages (337.8 ± 93.4 cells per mm^2^). This number doubles over the next two days (p = 0.036), and nerves collected at peak severity contain 678 ± 94.49 CD68^+^ cells per mm^2^. These quantitative findings are depicted by representative cross sections shown in Fig. 2b.

In addition to the immune cells that migrate into nerves during the course of EAN, the endothelial cells that form the blood nerve barrier (BNB) exhibit increased cell adhesion molecule expression and chemokine release^14,26^. We qualitatively assessed intercellular adhesion molecule (ICAM)-1 in longitudinal sections of sciatic nerves over the course of EAN. ICAM-1 was not detected in nerves from naïve animals. Staining co-localized with CD31 + endothelial cells was apparent at EAN onset and persisted through the effector phase and peak of disease severity (Fig. 2c).

### Permeation of a small molecule tracer over the course of EAN

**Fig. 3:**
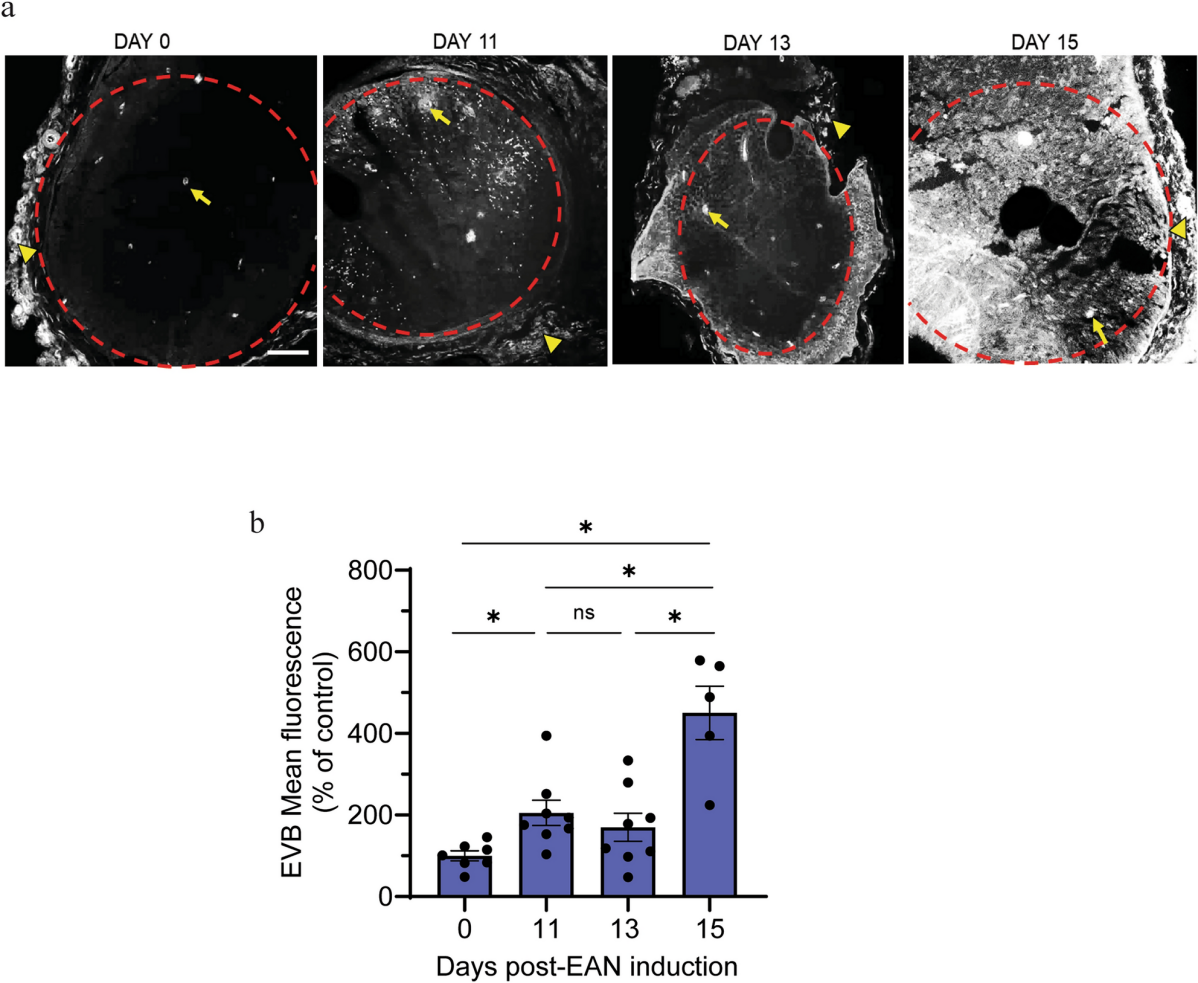
Permeation of a small molecule tracer over the course of EAN**.** Rats received a tail vein injection of Evans blue dye (EVB, 69 kDa) at key disease stages, and nerves were collected 30 min later. (**a**) Representative images demonstrating EVB permeation in nerve sections. Scale bar, 100 µm; arrow, endoneurial vessel; arrowhead, epineural vessel; dashed line delineates the endoneurial space. (**b**) Quantitative analysis of EVB permeation (λ_ex_/λ_em_ = 610/680 nm) in nerve sections at indicated days. Data shown are the mean ± SEM, n = 5–8 nerves (4 rats)/group, *p < 0.05, Ordinary one-way ANOVA followed by Fisher’s LSD multiple comparison.

We utilized Evans blue dye as a small molecule tracer to assess changes in vascular permeability over the course of EAN. Evans Blue binds to serum albumin at high affinity upon intravenous administration resulting in an approximate molecular weight of 69 kDa^27,28^. In control rats, the dye was restricted to the outer epineurium of nerve cross sections (arrowhead, Fig. 3a) and the lumen of endoneurial vessels (arrow, Fig. 3a). Further, more sections per animal exhibited epineural dye accumulation than endoneurial dye accumulation (Supplementary Fig. S1). While dye within endoneurial vessel lumen is apparent across all stages of disease (arrows), we observed a significant increase in dye permeation within the endoneurium at EAN onset (Day 11, 205.33 ± 30.9%) compared with naïve control rats (99.9 ± 12.01%, Fig. 3b). At peak severity (Day 15), endoneurial Evan’s blue dye permeation was at its maximum (450.268 ± 65.36%), a greater than four-fold increase over control (Fig. 3a, b). There was no difference in the frequency of epi- and endoneural accumulation at the peak of EAN severity (Supplementary Fig. S1).

### Nanoparticle PEGylation and characterization

**Fig. 4:**
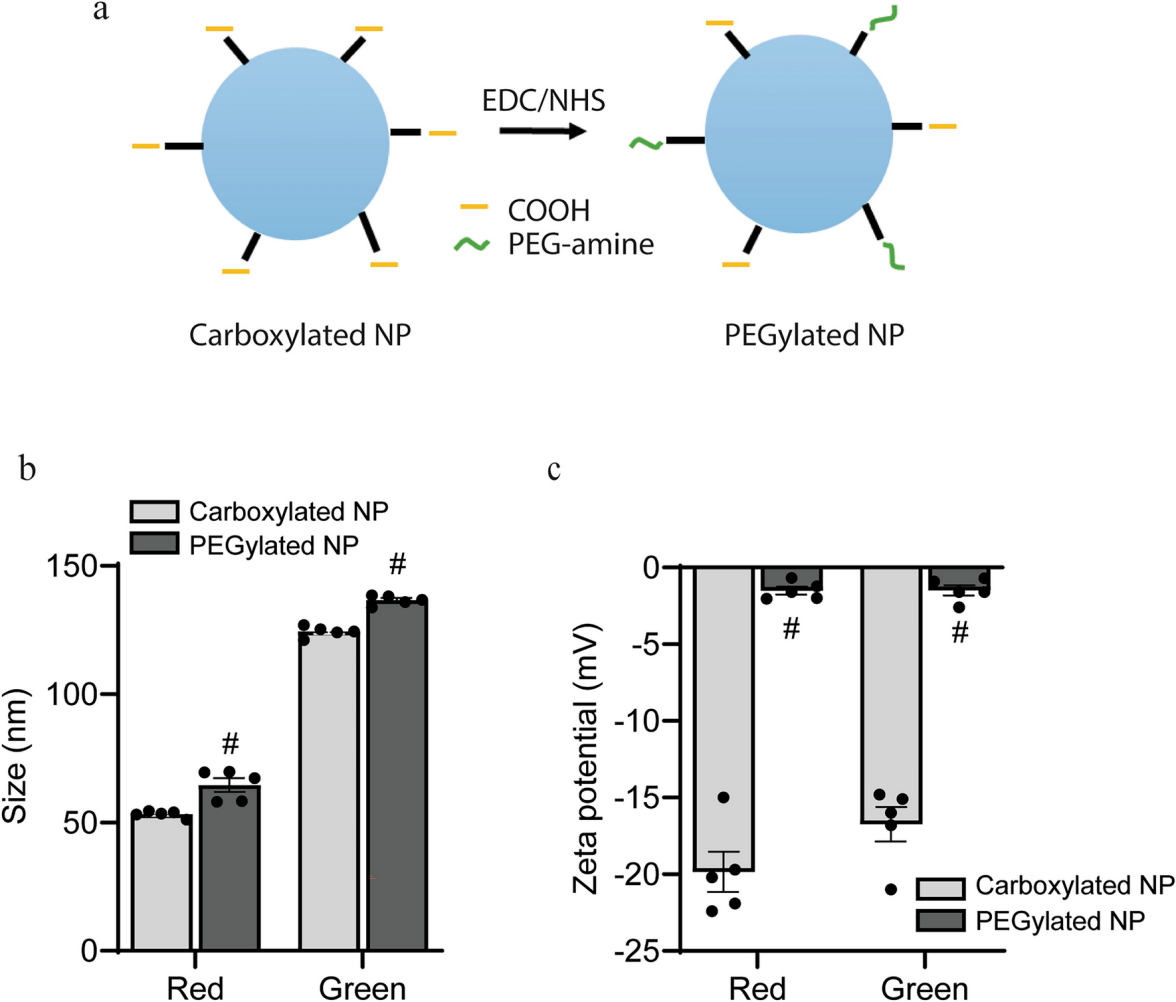
Modification and characterization of polystyrene nanoparticles. (**a**) Schematic demonstrating the covalent attachment of poly(ethylene) glycol (PEG)-amine to carboxylated NPs using carbodiimide chemistry (EDC/NHS). (**b**) Size (nm) and (**c**) zeta potential (mV) of carboxylated NPs and PEGylated NPs. Values shown are mean ± SEM, n = 5 separate batches, *p < 0.05 vs. carboxylated counterpart, multiple unpaired t-tests.

We investigated the accumulation of two sizes of polystyrene NPs corresponding with separate spectral wavelengths (λ_ex_/λ_em_ = 580/605 and λ_ex_/λ_em_ = 505/515). NPs were obtained as carboxylated FluoSpheres with nominal diameters of 40 nm and 100 nm, respectively (Table 1). For prolonged systemic circulation and reduction of protein opsonization^19^, we covalently attached mPEG-amine to carboxyl functional groups via carbodiimide chemistry (Fig. 4a). PEGylation was confirmed by an increase in NP hydrodynamic diameter and a shift in surface charge or zeta potential. NP size increased from 53.15 nm to 64.5 ± 2.7 nm (red) and from 124.3 nm to 136.5 ± 1.3 nm (green, Fig. 4b). This change in size with PEGylation was identical for both FluoSphere formulations and is in line with values in the literature^17,29^. Zeta potential of carboxylated NPs (− 18 mV) shifted to − 1.5 mV for both FluoSpheres (Fig. 4c).

**Table 1:** Nominal, measured, and PEGylated nanoparticle diameters.

	Nominal diameter (nm)	Measured diameter (nm)	PEGylated diameter (nm)
Red	40	53.15 ± 0.5	64.5 ± 2.7
Green	100	124.3 ± 0.9	136.5 ± 0.8

### Accumulation of nanoparticle tracers over the course of EAN

**Fig. 5:**
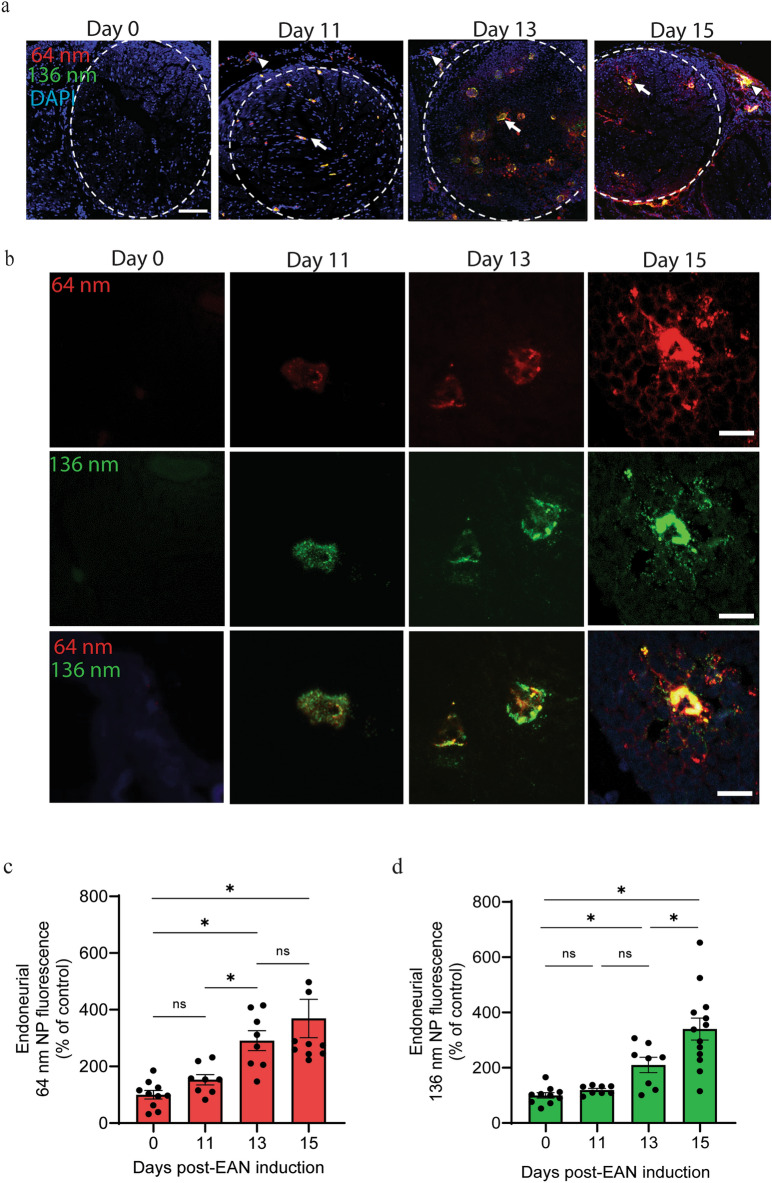
Accumulation of nanoparticle tracers in the endoneurium over the course of EAN. Rats received a tail vein injection of a cocktail of 64 nm (red) and 136 nm (green) PEGylated NPs at key disease stages, and nerves were collected 30 min later. (**a**) Representative 10 × images of transverse nerve sections. Scale bar = 100 µm; arrow, endoneurial vessel; arrowhead, epineural vessel; dashed line delineates the endoneurial space. (**b**) Fluorescence intensity in regions of interest around endoneurial vessels was assessed (Scale bar, 30 µm) and expressed as % increase over Day 0 control (**c**,**d**). Data shown are the mean ± SEM, n = 4–5 rat/group, *p < 0.05, Ordinary one-way ANOVA followed by Fisher’s LSD multiple comparison.

To investigate the potential of vascular permeability to facilitate delivery of NPs to peripheral nerves over the course of EAN, we administered PEGylated NPs of varying diameters and wavelength (64 nm, λ_ex_/λ_em_ = 580/605 and 136 nm, λ_ex_/λ_em_ = 505/515) and quantified distribution in sciatic nerve cross sections using confocal microscopy (Fig. 5). Representative 10 × magnification sciatic nerve cross sections (Fig. 5a) demonstrate both a lack of apparent epi- and endoneurial vessel accumulation in naïve animals (dashed line indicates delineation) as well as disease stage dependent increases in fluorescence intensity of NPs (Fig. 5a, b). Standardized regions of interest (ROIs) were selected around endoneurial vessels (indicated by arrows in Fig. 5a) for quantitative assessment of NP accumulation relative to respective control (Fig. 5b). Both NP sizes exhibited disease-stage dependent accumulation in nerves. Accumulation of 64 nm NPs within the endoneurium increased three-fold between onset and effector phase of EAN (to 290.54 ± 18.31%, p < 0.05), but the increase from the effector phase to peak severity was not significant (290.54 ± 18.31% and 390.07 ± 67.81% respectively, p = 0.2), Fig. 5c). In contrast, accumulation of 136 nm NPs within the endoneurium was not significantly increased over control at EAN onset (119.57 ± 4.98%), but accumulation increased significantly between the effector phase and peak severity EAN (from 210.05 ± 27.63% to 339.91 ± 39.57%, p < 0.05, Fig. 5d). Accumulation of NPs in the epineurium is apparent (arrowheads in Fig. 5a) over the course of EAN but is only significantly increased at peak severity of EAN (Fig. 5a, Supplementary Fig. S2).

**Fig. 6:**
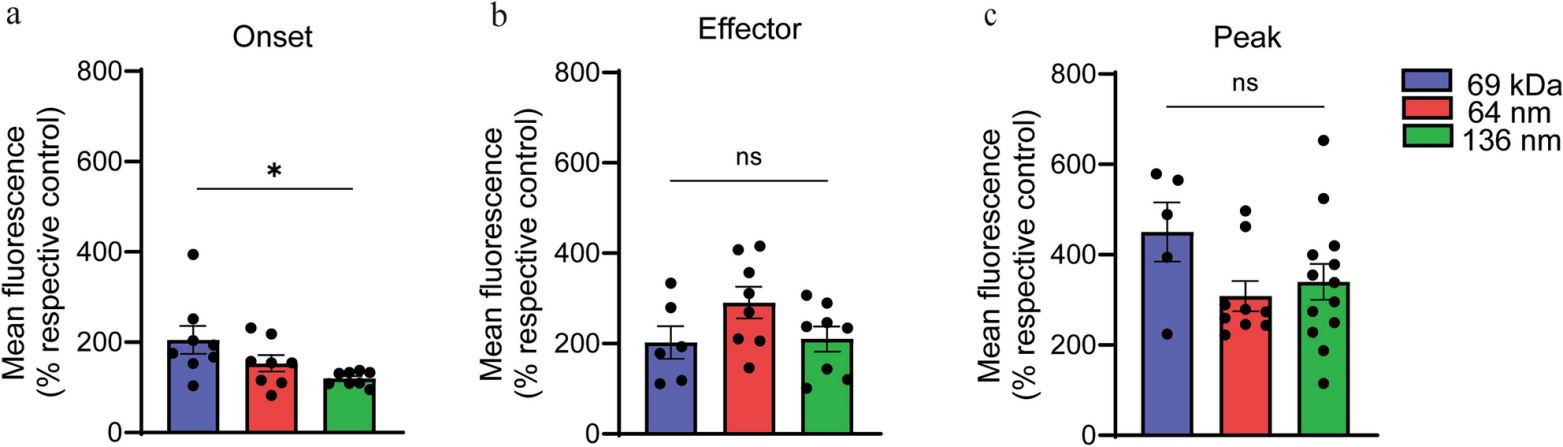
Analysis of size-dependent permeation over the course of EAN. Fluorescence of three different sized tracers in transverse sections of sciatic nerves collected were expressed as an increase over each tracers’ respective control (Day 0) and directly compared at (**a**) onset, (**b**) effector phase, and (**c**) peak severity of EAN. Data shown are the mean ± SEM, n = 4–5 rat/group, *p < 0.05, Ordinary one-way ANOVA followed by Fisher’s LSD multiple comparison.

We further compared accumulation of each tracer (small molecule and NP) at each stage of EAN (Fig. 6). At the disease onset, the increases in Evans blue associated fluorescence (234 ± 30.9%) were greater than those of the 64 nm and 136 nm NP tracers (153.1 ± 18.31%, 119.6 ± 5.57%), respectively (Fig. 6a). In the effector phase and peak severity EAN, no significant difference was observed between the accumulation of each of the three tracers (Fig. 6b, c).

### Analysis of NP accumulation in different regions of the nerve over the course of EAN

**Fig. 7:**
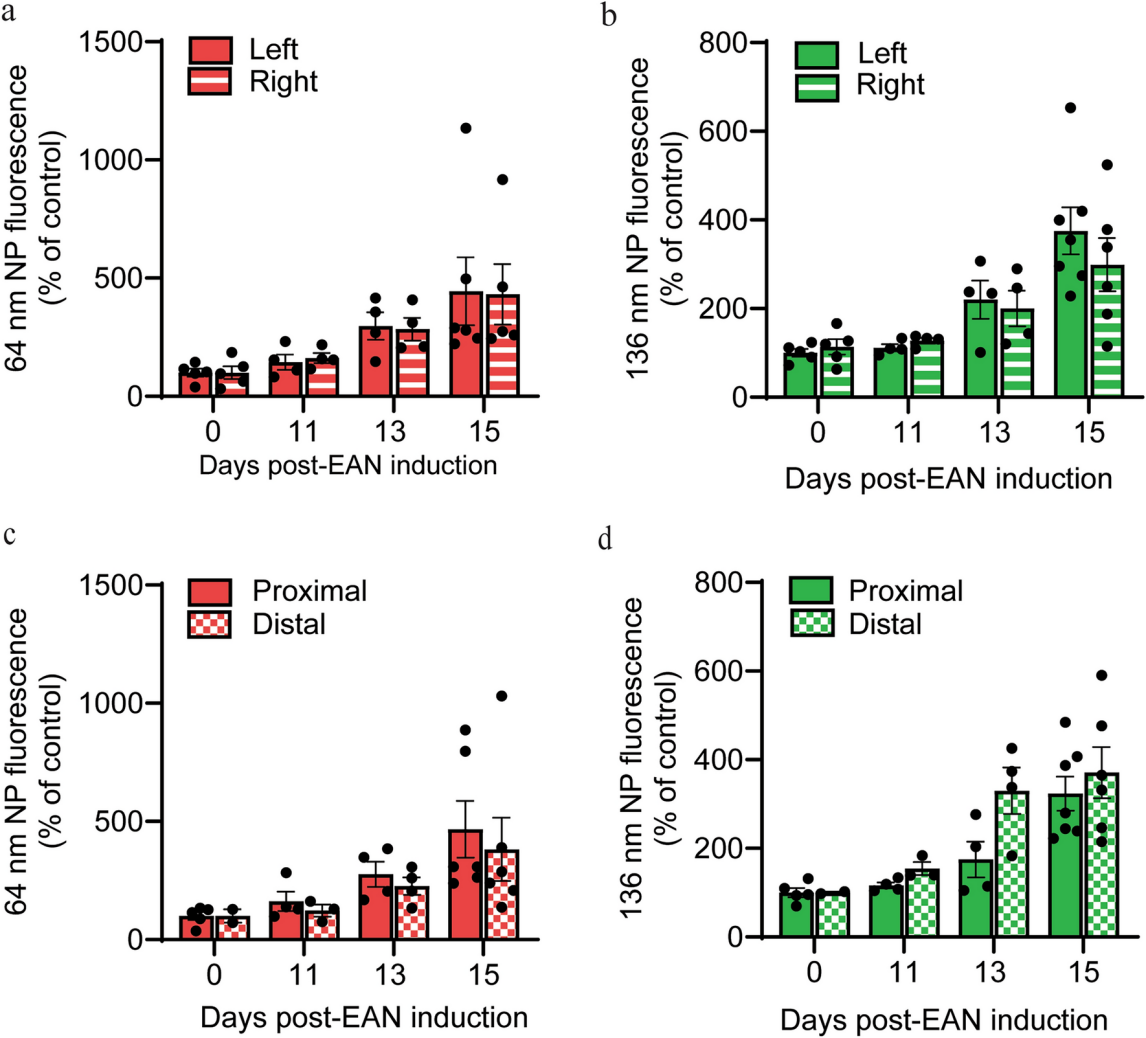
Spatial analysis of nanoparticle tracers over the course of EAN. Quantitative analysis of (**a**,**b**) lateral and (**c**,**d**) longitudinal distribution of 64 nm and 136 nm NP tracers, as indicated. Shown in (**a**,**b**) is a comparison between the nerve closest to the hind paw injection of CFA + antigen (ipsilateral, solid bars) and the contralateral nerve (patterned bars). Shown in (**c**,**d**) is a comparison between proximal (solid bars) and distal (patterned bars) regions of the nerves. All data shown are the mean ± SEM, n = 4–5 rats/group, multiple unpaired t tests.

Given that we induce EAN with a single injection of the antigen/adjuvant emulsion into the left hind footpad, and the injected foot exhibits local inflammation and edema over the course of disease, we sought to assess any lateral differences in distribution throughout the nerves. For both NP sizes, no significant difference was found between the accumulation in the left or right sciatic nerve over the disease course (Fig. 7a, b). Further, given potential regional variation in blood flow through the microcirculation, we assessed NP accumulation in proximal and distal regions of the sciatic nerves. No significant difference was found between either of these regions over the course of the disease (Fig. 7c, d).

### Correlation between NP accumulation and EAN clinical score

**Fig. 8:**
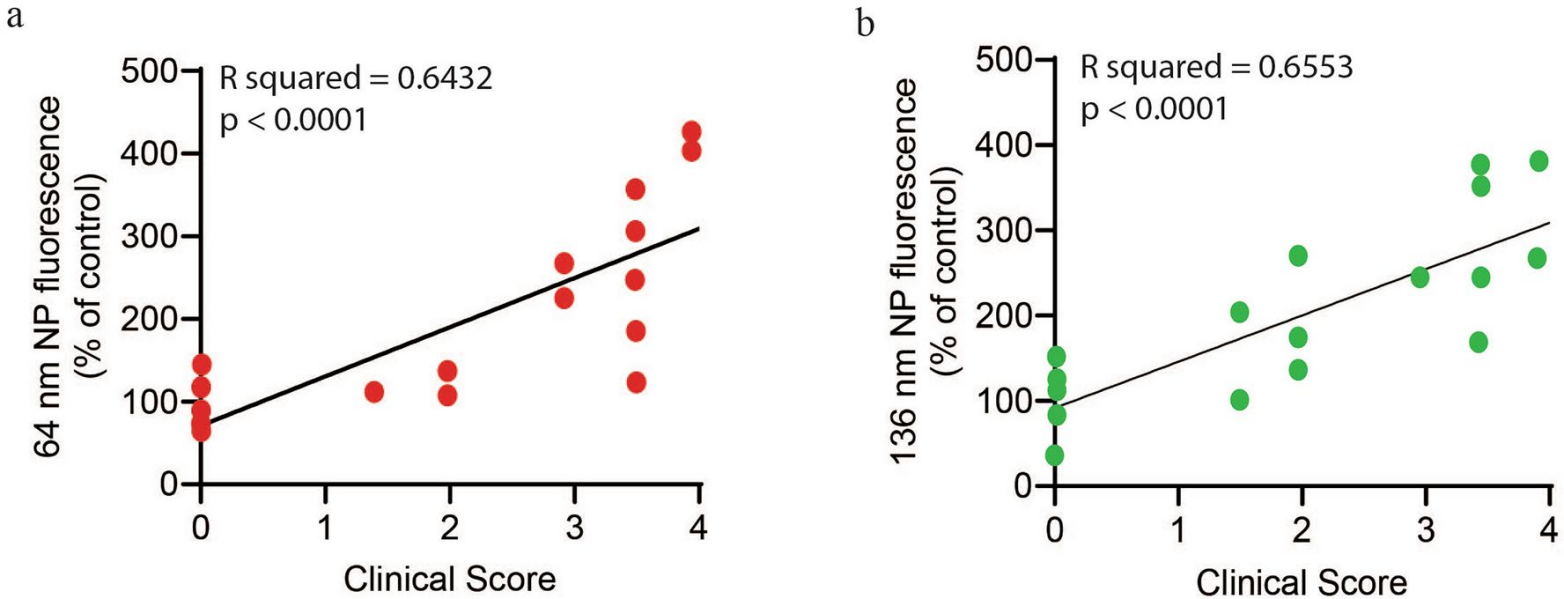
Correlative analysis of clinical score and NP accumulation. Dot plot showing positive correlation between clinical score and (**a**) 64 nm NP-associated fluorescence (**b**) 136 nm NP-associated fluorescence. Values plotted are for n = 16–17 rats.

We analyzed our data by day post-EAN induction at defined disease stages: onset, effector phase, and peak severity of EAN. It follows that clinical scores also increase over the course of EAN. To confirm our hypothesis that increased clinical scores are associated with increased fluorescence, and to extrapolate our findings to other EAN models that may exhibit onset or peak severity on different days, we analyzed the correlation between clinical score and NP accumulation. We found a strong positive correlation between clinical score and NP accumulation for both 64 nm (Fig. 8a) and 136 nm (Fig. 8b) NPs, suggesting that more severe clinical symptoms are associated with increased vascular permeability within the BNB.

## Discussion

The BNB represents a key obstacle in delivering therapeutic compounds to peripheral nerves under physiological conditions. Given advances in nanomedicine that have enabled targeted delivery strategies to locally inflamed sites^18^, including the inflamed or injured brain^16,17^, we hypothesized that EAN-associated pathology provides an opportunity to access the peripheral nerves for targeted delivery. Our work builds upon early EAN studies demonstrating nerve permeation of horseradish peroxidase^3,30^ and Evans blue dye^31^. While these studies were instrumental in identifying BNB permeability as a key pathological feature, the imaging data were not quantified and analyzed statistically over the course of disease. Further, while small molecule tracers can inform solute permeability, the approximate hydrodynamic diameters (~ 4 nm for Evans blue associated with serum albumin^32^) do not represent the dimensions of potential NP drug delivery systems^19^. Here, we assessed immune cell infiltration, adhesion molecule expression, and BNB permeability to small molecules and two distinct sizes of NP tracers at defined stages of disease progression. Our data demonstrate endothelial ICAM-1 expression and small molecule permeability at EAN onset. In the effector phase of EAN development, 64 nm NPs reach maximum accumulation in nerves, and at peak severity 136 nm NPs reach maximum accumulation. These findings indicate that inflamed nerves can be accessed from the systemic circulation for therapeutic delivery.

Many previous studies by different groups have demonstrated protective or therapeutic attenuation of EAN with systemically administered compounds spanning a range of anti-inflammatory or immunomodulatory mechanisms. Each of these studies has contributed to our understanding of EAN development and progression. However, the investigated therapies themselves are not feasible treatment options. Doses ranging from 40 to 300 mg/kg^8,10,33,34^, administered twice a day in some cases^8,9^, suggest poor bioavailability and significant risk of off-target effects and systemic toxicity^7,10,11,26^. While some examples, such as sphingosine-1-phosphate (S1P) inhibitors^26^ or peroxisome proliferator-activated receptor gamma (PPARγ) antagonists^35^, act systemically on immune organs and circulating immune cells, several examples act on sites within affected nerves. Given our findings that circulating NPs accumulate in affected nerves, PEGylated nanoformulations may represent a delivery strategy that allows for effective doses to be achieved at the desired sites.

Endothelial cells, macrophages, or Schwann cells within nerves may represent therapeutic targets that would benefit from NP delivery systems. Sarkey et al. found that lovastatin therapeutically attenuated EAN (25 mg/kg/day) by limiting immune cell trafficking into nerves^11^. Our subsequent work demonstrated that statins act directly on the BNB and that the therapeutic mechanism may involve inhibition of chemokine release from BNB endothelial cells^36,37^. The non-nitrogen containing bisphosphonate, clodronate, was shown to eliminate nerve macrophages and attenuate EAN when administered therapeutically after symptom onset^6^. While the S1P inhibitor fingolimod is primarily thought to attenuate EAN through peripheral immune modulation as described above^26^, it also reduces the number of apoptotic Schwann cells. The authors hypothesize that the lipophilic compound may diffuse into nerves and act directly on Schwann cells via S1P receptors to improve Schwann cell survival in EAN. Lysophosphatidic acid is another candidate with receptors on Schwann cells that been shown to attenuate EAN by modulating Schwann cell function and differentiation directly^38^.

Therapeutic targets associated with peripheral nerves may include axonal transcription factors or receptors at the nodes of Ranvier. Pitarokoili et al. demonstrated that dimethyl fumarate (DMF) reduces EAN severity by upregulating axonal levels of the transcription factor Nrf-2, leading to subsequent reduced demyelination and increased axonal survival^8^. DMF would benefit from a NP delivery system to increase bioavailability and nerve levels, given its poor water solubility and 45 mg/kg twice a day dosing. Thrombin receptors localized at nodes of Ranvier may also serve as potential therapeutic targets^39^. Thrombin activity increases during EAN, and activation of the axonal thrombin receptor protease activated receptor (PAR)-1 contributes to conduction blocks and destruction at the nodes of Ranvier. Administration of either a nonselective thrombin inhibitor (4.4 mg/kg/day) or a highly specific thrombin inhibitor (69.8 mg/kg/day) attenuated EAN. Given the role of thrombin inhibitors as anticoagulants, excessive bleeding was observed in animals’ post-mortem. Targeted delivery may alleviate this risk in GBS/EAN.

Additional therapeutic targets within peripheral nerves include sodium channels and cytoskeleton-associated motor proteins. The sodium channel blocker flecainide attenuated EAN when administered at 30 mg/kg twice daily starting after disease onset. The authors propose that by blocking sodium currents, flecainide prevents detrimental accumulation of sodium at sites of inflammation and prevents subsequent increases in axonal calcium ions. Sodium channel blockers are associated with arrhythmias and cardiac side effects^40^, which precludes systemic administration for GBS/EAN, particularly given the dosing for which beneficial effects on EAN were observed. The kinesin-5 inhibitor monastrol reduced EAN by enhancing neurite outgrowth within axons^7^. This therapy was administered at peak severity of disease, reflecting a later time when a patient would be diagnosed with GBS. Given the importance of axon regeneration after the effector phase and disease peak, monastrol represents an ideal candidate for later administration.

Despite clear size- and disease stage-dependent patterns in NP associated fluorescence, and a lack of lateral or longitudinal variation, NP accumulation appears to be centered upon endoneurial blood vessels (see Fig. 5). We focused on defined regions of interest around endoneurial blood vessels to quantify our data. There are multiple mechanisms by which circulating NPs can gain access to peripheral tissue from the circulation, including both transcellular and paracellular routes^41^. Transcellular routes may involve receptor mediated endocytosis or uptake via caveolae; however, PEGylated NPs are less likely to interact with endothelial cells in this manner^42^. In foundational studies of BNB integrity in EAN, Powell et al.observed separation of adjacent endothelial cells within nerves of affected rats, allowing luminal contents direct access to endoneurial space^43^. Dong et al.later demonstrated that leukocytes enter nerves during AIDP by crossing the BNB between endoneurial endothelial cells^44^. While these previous findings provide evidence that NP entry during EAN may occur in a paracellular manner, we are unable to conclude this from the current work. Future studies will overcome this limitation by colocalizing NPs with caveolae, plasmalemma vesicle-associated protein, and tight junction proteins to better elucidate the mechanisms by which NPs cross the BNB^45^.

The degree to which NPs can diffuse into the endoneurium has implications for the types of small molecules that would benefit from a NP delivery system. In the case of statins^11,24,36^, the therapeutic target (endothelial cells that form the endoneurial vessel wall) is clearly accessible via NPs interacting with the BNB from the circulation. Clodronate may also act closely to the BNB to eliminate macrophages. Axonal and Schwann cell targets require further diffusion of NPs into the endoneurium after crossing the BNB. Diffusion of NPs, and further diffusion of drug payload after release from NPs, has been demonstrated to occur on the order of millimeters after NP entry into the brain^46^. This suggests that within nerves, given the proximity of endoneurial vessels with axons and glia, simple diffusion may enable access to most sites within the endoneurium. Active targeting strategies may enable further penetration of NPs into the endoneurium^47–49^, and lipophilic payloads may diffuse more deeply into the endoneurium once released from polymeric NPs^50,51^.

We induce EAN with a single injection of the antigen/adjuvant emulsion into the left hind footpad, and the injected foot exhibits local inflammation and edema over the course of disease. Further, nerves exhibit regional variation in blood flow through the microcirculation as well as inflammation associated edema^52,53^. We assessed lateral and longitudinal distribution throughout the nerves to determine any regional variation in accumulation, and we did not observe any significant differences in NP accumulation between left and right or proximal and distal regions of the sciatic nerves. We did not assess permeation into nerve roots, given that the intermediate regions of the sciatic nerve are most restrictive to circulating molecules during homeostasis^13,54^. However, others have demonstrated that nerve roots exhibit increased vascular permeability earlier and at a greater magnitude than the sciatic nerve during EAN^31^, and we anticipate that nerve roots would promote NP accumulation no less than observed in sciatic nerves. Collectively, our findings confirm that inflammation in EAN is not localized to one region and is multifocal and widespread along the length of nerves. This supports the need for systemic, rather than local, therapeutic approaches.

For extrapolation of our findings to the investigations of systemically administered compounds discussed above, we must consider our defined disease stages in the context of other preclinical EAN studies. The clinical course of EAN in rat models induced with P2 peptide does not vary significantly between groups, with onset around Day 10 ± 1 and peak severity at Day 16 ± 1 ^5,24,35^. Elahi et al.^55^ induced “mild” and “severe” phenotypes by varying the concentration of CFA (1–3 mg/ml) and number of injection sites (hind foot pad ± base of tail). In the severe phenotype, EAN onset was on Day 8 ± 1. While we analyzed mean fluorescence intensity at different days post-induction, we observed a strong correlation between clinical score and fluorescence intensity (Fig. 8), suggesting that our findings can be applied to other EAN models, even if disease onset and peak severity are not at Day 11 and Day 15.

For future translational therapeutic studies, it is critical to also consider how our findings align with the course of GBS/AIDP in human patients. Most GBS patients reach peak neuropathy within four weeks of onset^56^, and diagnosis based on electrodiagnostic data is not possible until closer to peak disease^15^. Therefore, while early diagnosis occurs in some cases^15^, EAN onset does not directly correspond with GBS diagnosis. In EAN rats, we demonstrated that 64 nm NPs can access the endoneurium before clinical disease has peaked, during the effector stage between EAN onset and peak severity. Given that the time spent in each stage for patients is on the order of weeks, not days, we anticipate that both patients presenting with symptoms during the effector phase as well as patients receiving a diagnosis closer to peak severity of GBS would be candidates for nanomedicine approaches. Importantly, one EAN study demonstrated benefits of their systemically administered compound (monastrol) when it was administered after peak severity disease^7^. An intravenously administered delivery system will seamlessly incorporate into the ongoing standard of care for GBS which includes plasmapheresis and IVIg^1,2^, further supporting the translational relevance of nanomedicine. Additional study of BNB permeability in GBS patients, as well as continued investigation of NP fate throughout the body in EAN, is needed.

Our study has some limitations. As described above, we are limited in the conclusions we can draw regarding mechanisms of NP entry into the endoneurium and subcellular fate of NPs within the endoneurium. The polymeric NPs used in this study were commercially available polystyrene beads, chosen for their uniformity and stability. It will be critical for future work to assess distribution and fate of NPs that are drug-loaded and biodegradable^57^, which may introduce variability that requires additional controls and considerations. In addition, a detailed analysis of biodistribution was not performed in this work. Covalently attached labels, such as quantum dots in a range of fluorescent spectra, can better enable fate tracking at the organ and subcellular levels using confocal and transmission electron microscopy^51^ and will be important to include in future analyses. This current study provides foundational evidence for NP delivery to nerves over the course of EAN and supports continued investigation of nanomedicine for peripheral neuropathies.

## Conclusions

In this study, we characterized vascular permeability and cellular changes at the BNB over the course of EAN. We demonstrated a disease stage dependent increase in immune infiltrates, adhesion molecule expression, and permeation of a small molecule dye. We demonstrated that NPs with a hydrodynamic diameter of 64 nm can cross the BNB and accumulate in nerves during the effector phase of EAN, and NPs with a hydrodynamic diameter of 136 nm cross the BNB and accumulate in nerves at peak severity EAN. These findings establish that EAN associated pathology enables nerve targeting of circulating NPs and set the groundwork for further development of NP drug delivery strategies in diseases like inflammatory peripheral neuropathies that necessitate targeted, systemic administration.

## Materials and methods

### Materials

Carboxylated modified Fluospheres^™^, specifically 40 nm red (F8793, λ_ex_/λ_em_ = 580/605) and 100 nm yellow-green (F8803, λ_ex_/λ_em_ = 505/515), 20× borate buffer, glycine, and ICAM-1 antibody (MA5407) were purchased from Thermofisher Scientific (Waltham, MA). Evans blue dye, N-(3-Dimethylaminopropyl)-Nʹ-ethyl carbodiimide hydrochloride (EDC), N-Hydroxysuccinimide (NHS), Amicon Ultra centrifugal filters (10 and 100 kDa), and incomplete Freund’s adjuvant were purchased from Sigma-Aldrich (St Louis, MO, USA). Methoxy poly(ethylene) glycol (mPEG)-amine (2 kDa) was obtained from Creative PEGWorks (Chapel Hill, NC). Phosphate buffered saline (10× PBS) and mouse anti rat CD68 primary antibody was obtained from Bio-Rad (Hercules, CA). P2 peptide was obtained from the Peptide Synthesis Core Facility of the Simpson Querrey Institute, Northwestern University. Heat-inactivated Mycobacteria tuberculosis (strain H37Ra) was purchased from Becton, Dickinson, and Company (Sparks, MD).

### Nanoparticle PEG conjugation (PEGylation)

Carboxylated FluoSpheres^™^ were PEGylated with mPEG-amine using carbodiimide chemistry and previously published methods^17^. First, 500 μL of FluoSpheres (2% (yellow green) and 5% (red) suspension from the manufacturer) were washed once using Amicon centrifugal filters (10 kDa MWCO) at 14,000×*g* for 15 min. Next, mPEG-amine was added at 5× molar excess followed by NHS (6.5 mg), EDC (15.4 mg) and borate buffer (6 ml, pH 8) and allowed to stir for 3 h. The reaction was quenched with glycine (75 mg, 100 mM) for 30 min. PEGylated FluoSpheres, herein referred to as NPs, were washed and collected using Amicon centrifugal filters (100 kDa MWCO) and resuspended to original concentration in filtered 1× PBS for storage at 4 °C.

### NP characterization

Hydrodynamic diameter and zeta potential of NPs were quantitatively assessed with dynamic light scattering using Zetasizer Nano ZS90 (Malvern Panalytical). A suspension of NPs (0.1 mg/ml) was prepared in deionized water and transferred to a disposable polystyrene cuvette or a disposable capillary cell. Suspension was equilibrated for 3 min in the cuvette and measured at 90° angle. Each sample was measured four times.

### EAN induction

Experiments were conducted using protocols approved by the Edward Hines, Jr., VA Hospital Institutional Animal Care and Use Committee in accordance with the principles of laboratory animal care and in accordance with the ARRIVE (Animal Research: Reporting of In Vivo Experiments) guidelines 2.0. Adolescent male Lewis rats were housed in pairs, allowed free access to standard rat chow and water, and maintained on a 10/14 h light/dark cycle. Naïve rats (Envigo, Indianapolis, IN) were induced with EAN using P2 peptide as we have previously described^24^. Briefly, anesthetized rats (ketamine (100 mg/kg)-xylazine (5 mg/kg)) were injected with a freshly prepared emulsion containing (1:1 v/v) purified P2 myelin neuritogenic peptide (residues 53–78, 100 μg) suspended in sterile saline and Freund’s adjuvant supplemented with a final concentration of 5 mg/ml heat-inactivated Mycobacterium tuberculosis (strain H37RA) into one hind footpad. Control rats received a footpad injection of sterile saline. Weights and clinical progression of disease were assessed daily beginning on Day 7. Rats receive a score of 1 (loss of tail tone), 2 (disrupted gait), 3 (mild hemiparesis), 4 (severe paraparesis), or 5 (paraplegia), including increments of 0.5^24,25^.

### Intravenous injection of tracers

On the designated day, ketamine-anesthetized control and EAN rats received a single intravenous (tail vein) infusion of Evans blue dye (2% w/v suspended in PBS) or a cocktail of NPs (8 mg total/rat, suspended in diH_2_O). Time points for administration of Evans blue or NPs included EAN onset (Day 11), effector phase (Day 13) and peak severity EAN (Day 15).

### Tissue collection

Thirty minutes after infusion of tracers, rats were euthanized, and sciatic nerves were collected. Nerves were fixed for 4 h in 4% paraformaldehyde followed by incubation in 30% sucrose/PBS overnight at 4 °C. The next day, nerves were incubated in 50% optimal cutting temperature (OCT) medium/50% sucrose/PBS for 2 h followed by embedding in OCT and snap freezing in liquid nitrogen. Embedded tissue was stored at − 80 °C until further analysis.

### Immunohistochemistry and Evans blue accumulation

Serial 10 μm cross and longitudinal sections were prepared from nerves using a Leica CM 1850 Cryostat, and the content and distribution of CD68^+^ immune infiltrates was determined by immunohistochemistry as we have previously described^24^. Washed sections were blocked for 30 min at room temperature in PBS containing 5% (v/v) normal goat serum and 0.05% (v/v) Triton X-100 (blocking buffer). Cross sections were incubated with a 1:250 dilution of mouse anti-CD68 (macrophage marker, clone ED1, purified IgG primary antibody) followed by a 1:1000 dilution of Alexa Fluor 488 anti-mouse secondary antibody and coverslipped with prolong diamond antifade mountant containing DAPI. Infiltrating CD68^+^ macrophages were visualized on a Nuhsbaum inverted confocal microscope and semi-quantified in a blinded manner with ImageJ software. Longitudinal sections were incubated with a 1:100 dilutions of mouse anti-CD54 (ICAM-1, clone W3/25, purified IgG) and rabbit anti-CD31 (PECAM-1) primary antibodies followed by 1:1000 dilutions of Alexa Fluor 488 anti-mouse and Alexa Fluor 568 anti-rabbit secondary antibodies and similarly coverslipped. CD31^+^ and CD54^+^ cells were visualized as above, and endothelial ICAM-1 expression was qualitatively assessed.

Evans blue accumulation was assessed in the same sections at ×10 magnification at laser setting of 561 nm (photomultiplier tube (PMT) = 610–677 nm, 27.754% laser intensity). Z-stacked images of nerves were obtained and converted to one image using maximum projection on Leica LAS-X software. For assessment of endoneurial NP accumulation, five random regions of interest (ROI) were chosen in each cross-section (six nerve sections per animal, four animals per group) and fluorescence intensity was quantified using ImageJ software. Epineural NP accumulation was assessed qualitatively and is expressed as the number of sections out of 6 that exhibit staining.

### NP accumulation

Serial 10 μm cross sections were prepared from nerves using a Leica CM 1850 Cryostat and mounted onto Superfrost Plus microscope slides. Slides were incubated at room temperature for 30 min, washed once with PBS, and air dried. Coverslips were gently placed using Prolong Diamond Antifade mountant (Thermofisher Scientific). NP permeation was visualized using confocal microscopy. Images were captured at laser settings of 405 nm, 488 nm, 561 nm (PMT: 432–482 nm, 522–585 nm, and 610–677 nm) and laser intensity (17.07%, 21.35%, 27.74% respectively) was standardized for all nerve sections. For assessment of endoneurial NP accumulation, Z-stacked images (×40 magnification) of nerves were obtained and converted to one image using maximum projection on Leica LAS-X software. Standardized area ROIs around endoneurial blood vessels were chosen in the cross-section images (up to 10 nerve sections per animal, 4–5 animals per cohort) and fluorescence intensity was quantified using ImageJ software. For assessment of epineural NP accumulation, ×10 magnification images were obtained as above. Epineural accumulation was quantified as: (fluorescence intensity of ROI containing entire fascicle)–(fluorescence intensity of ROI containing endoneurium only) and normalized to control (Day 0) fluorescence.

### Statistics

All statistical analyses were conducted using GraphPad Prism 10.2.1 (397). Data are expressed as the mean ± SEM of N observations, and p < 0.05 was considered statistically significant. Non-significant observations are denoted with n.s. Statistical significance across multiple experimental groups (EAN score and weight, leukocytic infiltrates, Evans blue permeation, NP accumulation comparison at onset, effector phase, and peak severity of EAN) was conducted using one-way ANOVA followed by Fishers LSD and Tukey’s post-hoc test. Frequency of evans blue permeance in the epi- and endoneurium was assessed with a mixed effects analysis followed by Tukey’s post-hoc test. Statistical significance of paired groups was conducted using multiple unpaired t-tests. Statistical significance and R value of clinical score and NP accumulation was conducted using simple linear regression correlation analysis.

## Supplementary Information

Supplementary Information.

## Data Availability

Data sharing is not applicable to this article as no datasets were generated or analyzed during the current study.

## Abbreviations

GBS: Guillain-Barré syndrome
PNS: Peripheral nervous system
EAN: Experimental autoimmune neuritis
AIDP: Acute inflammatory demyelinating polyneuropathy
BNB: Blood nerve barrier
NP: Nanoparticle
EPR: Enhanced permeation and retention
EDC: N-(3-Dimethylaminopropyl)-Nʹ-ethyl carbodiimide hydrochloride
NHS: N-Hydroxysuccinimide (NHS)
mPEG: Methoxy poly(ethylene) glycol (mPEG)
PBS: Phosphate buffered saline
OCT: Optimal cutting temperature
IHC: Immunohistochemistry
CFA: Complete Freund’s adjuvant
ROI: Region of interest
ICAM-1: Intracellular adhesion molecule
PPARγ: Peroxisome proliferator-activated receptor gamma
DMF: Dimethyl fumarate
