# Supplementary material for: Blood nerve barrier permeability enables nerve targeting of circulating nanoparticles in experimental autoimmune neuritis
Source: Sci Rep. 2025 Apr 6;15:11763. doi: 10.1038/s41598-025-96231-z (PMC11973151; doi:10.1038/s41598-025-96231-z)
Supplement: Supplementary file 1 — Supplementary Information. [file 41598_2025_96231_MOESM1_ESM.pdf]

Supplementary information

Blood nerve barrier permeability enables nerve targeting of circulating nanoparticles in experimental autoimmune neuritis

Chanpreet Kaur, [1,2] Ellaina Villarreal, [1] Maleen H. Cabe,[1,2] and Kelly A. Langert* [1,2]

Department of Molecular Pharmacology and Neuroscience

Loyola University Chicago, Stritch School of Medicine, Maywood, IL 60153 (U.S.A.) [1]

Research Service

Edward Hines Jr. VA Hospital, Hines, IL 60141 (U.S.A.) [2]

Supplementary Fig. S1.

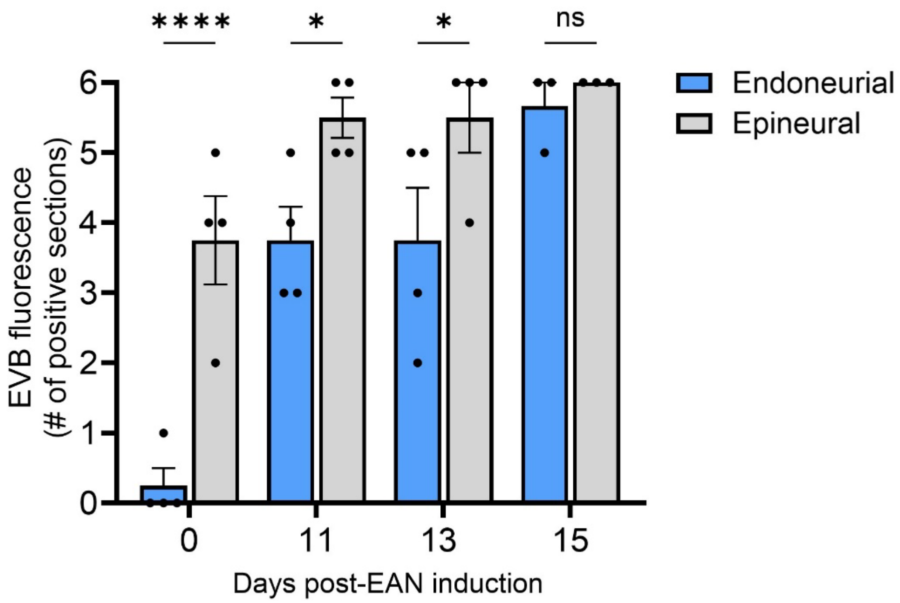

Supplementary Fig. S1. Accumulation of a small molecule tracer in the epineurium and endoneurium over the course of EAN**.** Rats received a tail vein injection of Evans blue dye (EVB, 69 kDa) at key disease stages, and nerves were collected 30 minutes later. EVB permeation ($\lambda_{ex}/\lambda_{em}$ = 610/680 nm) in epi- and endoneurial compartments of transverse nerve sections was assessed. Data are reported as the number of nerve sections (out of 6 per rat) exhibiting positive staining in the respective compartment. Shown are the mean ± SEM, n=3-4 rats/group, ****$p<0.0001$, *$p<0.05$, Mixed effects analysis followed by Tukey's multiple comparison.

Supplementary Fig. S2

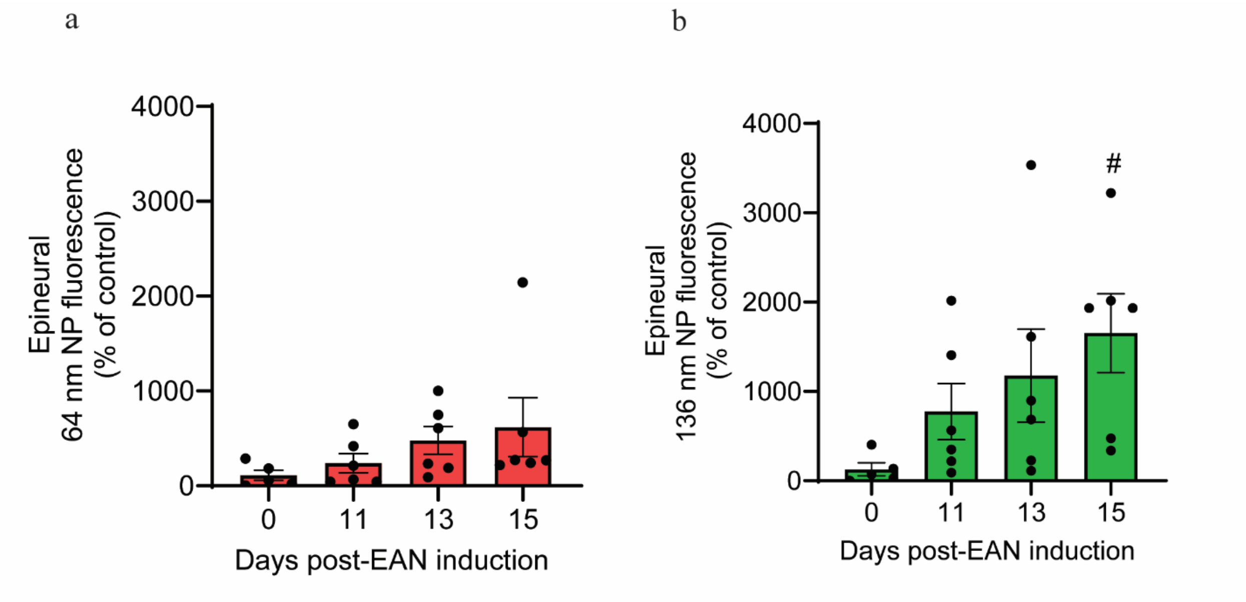

Supplementary Fig. S2. Accumulation of nanoparticle tracers in the epineurium over the course of EAN**.** Rats received a tail vein injection of a cocktail of **(a)** 64 nm and **(b)** 136 nm PEGylated NPs at key disease stages, and nerves were collected 30 minutes later. Epineural accumulation was quantified as: (fluorescence intensity of ROI containing entire fascicle) – (fluorescence intensity of ROI containing endoneurium only) and normalized to control (Day 0) fluorescence. Data shown are the mean ± SEM, n= 4-5 rat/group, #$p<0.05$ vs. Day 0, ordinary one-way ANOVA followed by Fisher's LSD multiple comparison.
